# Investigating the Role of African Horse Sickness Virus VP7 Protein Crystalline Particles on Virus Replication and Release

**DOI:** 10.3390/v14102193

**Published:** 2022-10-04

**Authors:** Shani Bekker, Christiaan A. Potgieter, Vida van Staden, Jacques Theron

**Affiliations:** 1 Department of Biochemistry, Genetics and Microbiology, University of Pretoria, Hatfield 0083, South Africa; 2 Deltamune (Pty) Ltd., 3 Bauhinia Street, Unit 34 Oxford Office Park, Highveld Techno Park, Centurion 0169, South Africa; 3 Human Metabolomics, Faculty of Natural and Agricultural Sciences, North-West University, Potchefstroom 2520, South Africa

**Keywords:** AHSV, equine, VP7, crystalline particles, reverse genetics, replication, virus release, cytopathic effect, pathogenesis

## Abstract

African horse sickness is a deadly and highly infectious disease of equids, caused by African horse sickness virus (AHSV). AHSV is one of the most economically important members of the *Orbivirus* genus. AHSV is transmitted by the biting midge, *Culicoides*, and therefore replicates in both insect and mammalian cell types. Structural protein VP7 is a highly conserved major core protein of orbiviruses. Unlike any other orbivirus VP7, AHSV VP7 is highly insoluble and forms flat hexagonal crystalline particles of unknown function in AHSV-infected cells and when expressed in mammalian or insect cells. To examine the role of AHSV VP7 in virus replication, a plasmid-based reverse genetics system was used to generate a recombinant AHSV that does not form crystalline particles. We characterised the role of VP7 crystalline particle formation in AHSV replication in vitro and found that soluble VP7 interacted with viral proteins VP2 and NS2 similarly to wild-type VP7 during infection. Interestingly, soluble VP7 was found to form uncharacteristic tubule-like structures in infected cells which were confirmed to be as a result of unique VP7-NS1 colocalisation. Furthermore, it was found that VP7 crystalline particles play a role in AHSV release and yield. This work provides insight into the role of VP7 aggregation in AHSV cellular pathogenesis and contributes toward the understanding of the possible effects of viral protein aggregation in other human virus-borne diseases.

## 1. Introduction

African horse sickness (AHS) is a severe and fatal disease of equids that is caused by African horse sickness virus (AHSV). AHS has been classified as a notifiable OIE-listed disease, resulting in the implementation of significant constraints on international trade and increased disease surveillance [1]. AHSV has nine serotypes and is endemic to central Africa, spreading regularly to southern Africa and occasionally northern Africa [2,3,4]. AHSV is transmitted to the host via the *Culicoides* biting midge and therefore the global and seasonal distribution of AHSV is dependent on climate conditions of this insect vector [5,6].

Belonging to the *Orbivirus* genus and part of the *Reoviridae* family, African horse sickness virus (AHSV) is a complex non-enveloped virus with a characteristic double-stranded (ds) RNA genome consisting of ten segments contained within an icosahedral core particle that is encapsidated by an outer protein layer [7,8,9,10]. These segments encode seven structural proteins VP1–VP7, as well as four non-structural proteins: NS1, NS2, NS3/NS3A and NS4 [8,10,11,12]. The non-structural proteins are found in virus-infected host cells and are thought to play roles in virus assembly, transport and exit from the cell. The seven structural proteins make up the double-layered capsid, composed of an outer capsid and an inner icosahedral core particle [13,14,15].

During replication, certain hallmarks of infection start to appear such as the formation of structures and protein inclusions within the cytoplasm. NS1 self-assembles into tubule structures of unknown function within the cytoplasm of AHSV-infected cells [16,17]. These NS1 tubules have been shown to promote viral protein translation of a closely related orbivirus, bluetongue virus (BTV) [18] and NS1 has been reported to be involved in virus egress and morphogenesis [19]. Other particulate structures documented exclusively during AHSV infection include the formation of flat, hexagonal crystalline particles formed by AHSV VP7 [20]. The formation of these crystals is unique to AHSV among members of the *Orbivirus* genus and their functional significance, if any, remains to be discovered and is the focus of this study. AHSV VP7 is highly conserved among all nine serotypes [21]. To date, AHSV VP7 crystalline particles have been observed in cells infected with AHSV serotypes 3, 4 and 9 [20,22,23]. These crystals have been observed in both mammalian cells and a *Culicoides*-derived insect cell line (KC cells) infected with AHSV [22,24]. The fine structure of these flat crystals of AHSV VP7 were revealed to be highly ordered hexagonal lattices [20]. Another notable hallmark of orbivirus infection is the formation of virus inclusion bodies (VIBs), directed by non-structural protein NS2. These VIBs act as sites for virus replication and early virus assembly by recruiting the viral RNAs and proteins required for genomic packaging, replication and core assembly [25,26,27]. Major core proteins VP3 and VP7 self-assemble to encapsidate the dsRNA genome and transcriptase complex within these VIBs to form the core particle.

Once assembled, the core particles are released from VIBs and join the host exocytosis pathway with the aid of NS3/NS3A. It is during this virus egress stage that the outer capsid proteins VP2 and VP5 are incorporated onto the core particle to form a mature virion which then exits the cell via further NS3/NS3A facilitation [28,29,30,31]. The mechanism of BTV/AHSV virion exit from the cell depends on the cell type. Generally, in insect host cells, virus particles exit through the plasma membrane in a non-lytic manner allowing them to acquire a temporary membrane and allowing the host cell membrane to remain intact [22,32]. In mammalian cells, virus particles can be released in a lytic manner by disruption of the plasma membrane, resulting in morphological changes that eventually lead to cell death or cytopathic effect (CPE) [33,34].

Currently, the only method of protection against the virus is a polyvalent live-attenuated vaccine that confers broad protection against all nine AHSV serotypes. With the recent developments in reverse genetics technology, it has become possible to produce recombinant viruses containing defined mutations and/or markers in their viral genomes resulting in the rapid production of safe attenuated and “Differentiation of Infected from Vaccinated Animals” (DIVA)-compliant vaccines, respectively [35,36,37]. This strategy also allows the generation of Entry Competent Replication Abortive (ECRA), previously referred to as Disabled Infectious Single Cycle (DISC), vaccine strains such as the replication-defective VP6 deletion mutant virus strain [38,39,40]. These vaccines overcome the concern of reversion to virulence as implicated in attenuated vaccines, as they cannot replicate.

AHS can cause up to 95% mortality in susceptible horses [3]. The outcome of the disease is varied and depends on a variety of viral and host factors. Investigating the contribution of AHSV proteins to virulence is therefore a topic of great importance [41]. It has been previously suggested that the hallmark AHSV VP7 crystalline particles present in infected cells could possibly contribute to AHSV cellular pathogenesis [42]. Many viruses induce cellular remodelling during infection which results in the formation of such insoluble aggregates or inclusions that usually contain viral structural proteins [43]. Protein aggregation and the assembly of aggregates into supramolecular structures is extensively documented in many systems and has been shown to have a large impact on cellular biology and cause of disease [44,45,46].

Several viruses have been shown to cause neurodegeneration in humans due to protein aggregation in the brain [47,48,49]. Furthermore, viruses have suggested to be linked to the onset of protein aggregation-associated diseases such as SARS-CoV-2 and influenza A in Parkinson’s disease [50], and SARS-CoV-2 and herpes simplex virus in Alzheimer’s disease [51,52,53]. In vitro studies have recently revealed that SARS-CoV-2 ORF8b was found to form insoluble intracellular aggregates that caused cell death of epithelial cells by inducing ER stress and activation of the autophagy-lysosome pathway [54]. Furthermore, subcellular aggregation of SARS-CoV-2 ORF8 was detected in lung epithelial cells [55] further demonstrating that accessory proteins of pathogenic coronaviruses play a role in disrupting antiviral immune responses [56,57,58]. The SARS-CoV-2 spike protein peptides, NSP6 peptide, ORF10 protein and the NSP11 protein have also been shown to form aggregates in vitro and NSP11 aggregates are toxic to mammalian cell cultures [59]. Other examples include those virus proteins that are known to form amyloid aggregates which are implicated in the viral pathogenesis such as the influenza A PB1 [60,61,62] and nuclear export proteins [63].

Given that AHSV pathogenicity and disease is more severe than that of BTV [64], it is possible that the AHSV VP7 crystalline particles contribute to AHSV cellular damage and disease. In this study, we examined the role of AHSV VP7 crystalline particles on AHSV replication kinetics. We have recently reported on the discovery of seven amino acid substitutions that convert AHSV VP7 into a fully soluble protein, which does not self-assemble into the characteristic crystalline particles and is homogenously distributed throughout the cell when expressed in vitro [42]. It was also found that this soluble version of AHSV VP7 could interact with VP3 and self-assemble into empty core-like particles (CLPs) more efficiently than wild-type AHSV. Furthermore, sequence analysis revealed that crystalline particle formation is likely conserved among all nine AHSV serotypes [42].

In this study, we set out to use this soluble version of AHSV VP7 to study the impact of VP7 crystalline particle formation on virus replication by using a plasmid-based reverse genetics system to generate recombinant AHSV that does not form VP7 crystalline particles. Using the backbones of virulent (AHSV5) and attenuated (AHSV4) serotypes, we replaced wild-type segment 7 with a gene encoding our soluble mutant VP7, and used these recombinant viruses to study the effect of crystalline particle formation on virus yield and release in mammalian and insect cells. The interaction of soluble VP7 with other AHSV proteins during the replication cycle was also examined. The results obtained here allowed us to further understand the role of AHSV VP7 on replication, and possibly cellular pathogenesis. This study sheds light on the potential role of viral protein inclusions in disease.

## 2. Materials and Methods

### 2.1. Cells and Viruses

BSR-T7/5 cells are a clone of baby hamster kidney-21 cells that constitutively express T7 polymerase, and were used with permission from Ulla Buchholz, Department of Clinical Virology, Federal Research Centre for Virus Diseases of Animals, Tubingen, Germany [65]. The cells were cultured in Eagle’s minimum essential medium supplemented with Earle’s Balanced Salt Solution; 2 mM L-Glutamine (HyClone™, Logan, UT, USA), 1% non-essential amino acids (Sigma-Aldrich^®^, Merck, Darmstadt, Germany), 5% foetal bovine serum (FBS, Gibco^®^, Thermo Fisher Scientific, Waltham, MA, USA) and 1% penicillin and streptomycin (Lonza, Basel, Switzerland). Geneticin (Thermo Fisher Scientific, Waltham, MA, USA) was added every third passage at 1 mg/mL. Cells were grown at 37 °C in the presence of 5% CO_2_. KC cells (derived from *Culicoides variipennis*) were obtained from the Onderstepoort Veterinary Institute (Pretoria, South Africa) and cultured at 28 °C in TC-100 insect medium (Lonza, Basel, Switzerland) with nonessential amino acids supplemented with 10% FBS (Gibco^®^, Thermo Fisher Scientific, Waltham, MA, USA), 1% penicillin and streptomycin (Lonza, Basel, Switzerland), and antifungals (Fungizone, Sigma-Aldrich^®^, Merck, Darmstadt, Germany).

### 2.2. Plasmid-Based Reverse Genetics System

A plasmid-based reverse genetics system described previously [66] was used to recover wild-type and mutant viruses. Rescue of recombinant viruses involves the simultaneous transfection of ten transcription plasmids and six expression plasmids into BSR-T7/5 cells. To generate transcription plasmids for rescue of recombinant wild-type serotype 4 (rAHSV4) and serotype 5 (rAHSV5) viruses, the cDNA of each of the 10 complete AHSV segments for AHSV4 (accession numbers KM820849 to KM820858) and AHSV5 (accession numbers KM886344 to KM886353) were cloned into the pSMART-T7 transcription vector under the control of the phage T7 promoter with a HDV Ribozyme at the 3′ end (GenScript, Piscataway, NJ, USA). The six expression plasmids were generated by cloning the ORFs for AHSV5 VP1 (GenBank KM886344), VP3 (GenBank KM886346), VP4 (GenBank KM886347), VP6 (GenBank KM886352), VP7 (GenBank KM886350) and NS2 (GenBank KM886351) into the phCMV-Dream vector kindly provided by Prof PA van Rijn, Wageningen Bioveterinary Research, The Netherlands as described previously [66].

### 2.3. Construction of Transcription Plasmids That Encode Soluble AHSV VP7

To construct reverse genetics plasmids that encode the mutant soluble VP7 (sVP7), the In-Fusion cloning strategy [42] was used to introduce mutations into segment 7 (S7) cDNA. To construct AHSV4-sVP7 transcription plasmid, the plasmid vector pJAD-S7 [67], containing a cDNA copy of AHSV4 S7 (GenBank KM820855) was used as template. A dsDNA fragment of the VP7 backbone (position 827 to 1017) containing the mutations that encode the seven amino acid substitutions (underlined in gBlock sequence) responsible for converting AHSV VP7 into a soluble protein, i.e., P276H; R328A; V333N; A334P; P335M; V336P; and Q338P [42] was designed and synthesised using gBlock Gene Fragments (Integrated DNA Technologies, Coralville, IA, USA) (5′-ATGCGTATGTCTCTCACACTTGGCACGCATTACGCGCTGTCATTTTTCAGCAGATGAATATGCAGCCTATTAATCCGCCGATTTTTCCACCGACTGAAAGGAATGAAATTGTTGCGTATCTATTAGTAGCTTCTTTAGCTGATGTGTATGCGGCTTTGAGACCAGATTTCGCGATGAATGGTGTTAATCCGATGCCAGGGCCGATTAACAGAGCTCT-3′). This dsDNA fragment was inserted into the pJAD-S7 backbone using the In-Fusion^®^ HD Cloning kit (Clontech^®^, Takara Bio Inc., Shiga, Japan) cloning strategy. The dsDNA fragment was designed to include 15 bp homologous ends to the VP7 sequence and inserted into the VP7 plasmid backbone linearized by inverse PCR using primers that contained the same 15 bp homologous ends in their 5′ regions by In-Fusion^®^ reaction according to manufacturer’s instructions. The sequence of the resulting plasmid, pJAD-S7-276, was confirmed by Sanger sequencing.

To construct the AHSV5-sVP7 transcription plasmid, the pSMART-T7 transcription vector containing a cDNA copy of AHSV5 S7 (GenBank KM886350) was used as a template. A dsDNA fragment containing the mutations that encode for sVP7 (underlined in sequence) in the AHSV5 backbone was synthesised by GenScript (Piscataway, NJ, USA) (5′-ATGCGTATGTTTCGCACACCTGGCACGCCCTACGCGCAGCCATCTTTCAACAGATGAATATGCAGCCAATTAATCCGCCAATTTTTCCACCAACAGAAAGAAATGAGATTGTTGCATATTTGCTAGTAGCTTCTTTGGCAGACGTGTATGCGGCTTTAAGACCAGACTTCGCGATGAATGGTGTTAATCCGATGCCAGGGCCGATCAACAGAGCTCT-3′) and inserted into the S7 pSMART-T7 transcription vector backbone using In-Fusion^®^ HD Cloning kit as described above. The sequence of the resulting plasmid, pSMART-T7-sVP7, was confirmed by Sanger sequencing.

### 2.4. Rescue of Recombinant African Horse Sickness Viruses

Recombinant viruses were rescued by plasmid-based reverse genetics system as described previously [66,68]. Monolayers of BSR-T7/5 cells (~70% confluency in T25 flasks) were transfected with 5 µg of an equimolar mix of expression plasmids, combined with 5 µg of an equimolar mix of pSMART-T7 transcription plasmids, using Lipofectamine 2000 (Thermo Fisher Scientific, Waltham, MA, USA) according to manufacturer’s instructions. After 24 h incubation at 37 °C, cells were passaged once and harvested when 100% CPE was visible. Supernatant containing rescued virus was stored at 4 °C, and viruses were designated rAHSV4, rAHSV5, rAHSV4-sVP7 and rAHSV5-sVP7. Presence of recombinant virus was confirmed by immunofluorescence microscopy. Rescued virus was diluted in series and 200 µL of serial dilutions were used to infect BSR monolayers in a 24-well tissue culture plate. Cells were incubated for 24 h at 37 °C and fixed with 1:1 methanol:acetone. Cells were labelled with anti-VP7, anti-VP2, anti-VP5 and anti-NS2 antibodies and nuclei stained with DAPI as described in Section 2.5. Slides were analysed on a fluorescence microscope. Positive staining confirmed presence of live virus. Soluble VP7 distribution in rAHSV4-sVP7 and rAHSV5-sVP7 recombinant viruses was also confirmed. Virus titres were determined by endpoint dilution in BSR cells and expressed as TCID_50_/mL (50% tissue infective dose per millilitre) [69] and converted to pfu/mL by multiplying by 0.7.

### 2.5. Immunofluorescence Microscopy

Immunofluorescence microscopy was performed as previously described [70]. Cell monolayers were grown to ~80% confluency on coverslips in 24-well plates. BSR monolayers were infected with recombinant AHSV virus at a MOI of 0.1 and incubated at 37 °C for 48 h before being fixed in 4% (*w*/*v*) paraformaldehyde/PBS for 30 min at room temperature (RT). After fixation, cover slips were incubated for 15 min at RT in 0.5% Triton X-100/PBS to permeabilize the cells. BSR cells were blocked in 5% blocking solution (5% milk powder in PBS) directly after fixation followed by incubation with primary antibodies for 1 h. The cells were washed three times with wash buffer (0.5% (*v*/*v*) Tween20 in PBS) and incubated for 1 h with appropriate secondary antibody. Cells were washed and stained with 10 μg/mL DAPI (4′,6-Diamidine-2′-phenylindoledihydrochloride) in 1% blocking solution for 10 min at RT. Coverslips were mounted onto glass slides using Vectashield Mounting Medium (Vector Laboratories, Newark, CA, USA). Slides were then viewed using a Zeiss LSM 880 Laser Scanning Confocal Microscope (LSCM) coupled to an Airyscan detector. Images were processed and analysed for colocalisation by using Zeiss ZEN 3.3 Blue edition software package (Carl Zeiss Microscopy GmbH, Jena, Germany).

### 2.6. Antibodies

For the identification of AHSV VP7 and NS2 proteins, anti-VP7 guinea pig [71] and anti-NS2 rabbit [27] sera were available. All sera were preadsorbed with an uninfected BSR cell lysate to remove non-specific binding. VP2 and NS1 were labelled with AHSV4 VP2 and NS1 mouse monoclonal antibodies (Eurofins Technologies Ingenasa, Madrid, Spain). Primary antibodies were diluted 1:100 in 1% blocking solution (1% milk powder in PBS). For detection of labelling, Alexa Fluor 488 anti-guinea pig (green); Alexa Fluor 647 anti-rabbit (red); and Alexa Fluor 594 anti-mouse (red) conjugated antibodies (Thermo Fisher Scientific, Waltham, MA, USA) were used as secondary antibodies at a dilution of 1:250 in 1% blocking solution for LSCM.

### 2.7. Virus Growth Kinetics

Virus growth kinetics was carried out in BSR and KC cells. Cells were grown to ~80% confluency in 24-well plates and each well was infected with appropriate virus at a MOI of 0.1, in triplicate, and incubated at 37 °C (BSR) or 27 °C (KC cells). After 1 h, BSR cells were washed and media replaced with 0.7 mL complete media to remove unbound virus. Cells were incubated for a further 12, 24, 36, 48 or 72 h. At each time point, both released and cell-associated virus were harvested separately. The supernatant of each well was carefully collected (representing released virus fraction) and the remaining cells harvested in 0.7 mL clean media by scraping cells and homogenizing through a 22G needle several times (representing cell-associated virus fraction). Debris was removed by centrifugation at 2500 rpm for 10 min and virus fractions stored at 4 °C. Virus titre was determined by endpoint dilution in BSR cells for each triplicate fraction, i.e., cell-associated virus, released virus, or total virus. Briefly, cells were infected with 10-fold dilutions of each sample (6 repeats), plates were sealed, incubated for 72 h and wells were scored according to presence or absence of visible CPE observed with an inverted light microscope. Titres were expressed as TCID_50_/mL (50% tissue culture infective dose per millilitre) and converted to pfu/mL by multiplying by 0.7 [69]. All experiments were independently repeated 2 to 3 times. Results were expressed as means ± standard error of the mean. Unpaired Student’s *t*-test was applied to statistical differences amongst the wild-type and mutant groups for each time point separately. In all the experiments, *p* ≤ 0.05 was considered statistically significant with the following denotations used: * (*p* ≤ 0.05) and ** (*p* ≤ 0.01).

## 3. Results

### 3.1. Generation of Recombinant AHSV That Express a Soluble VP7 by Reverse Genetics

To understand the role of AHSV VP7 crystal formation during AHSV replication, we aimed to generate recombinant ‘synthetic’ AHSV that contains a modified segment 7 that encodes a soluble VP7 protein (sVP7), by reverse genetics. To also determine whether AHSV VP7 crystalline particles could play a role in virulence or cellular pathogenesis, this study set out to generate both recombinant AHSV4 (attenuated) and AHSV5 (virulent) viruses that do not form VP7 crystalline particles, i.e., rAHSV4-sVP7 and rAHSV5-sVP7, to compare their properties with their wild-type counterparts. Therefore, four recombinant viruses (i.e., rAHSV4; rAHSV4-sVP7; rAHSV5; and rAHSV5-sVP7) were generated by reverse genetics technology.

A plasmid-based reverse genetics system was used to recover wild-type and mutant viruses. Here, recombinant AHSV4 (rAHSV4), representing wild-type AHSV serotype 4 virus, was derived using segments 1–10 representing the live attenuated AHSV4 strain known as “LP” (large plaque strain attenuated from the OIE reference strain of AHSV4, HS 32/62). This avirulent AHSV4 LP strain is used as a vaccine in South Africa and its origin has been described previously by Baltus Erasmus [72]. Recombinant AHSV4 containing a mutated segment 7 that encodes a soluble viral protein 7 (rAHSV4-sVP7) was derived by using segments 1–6 and 8–10 of AHSV4 (LP) and substituting S7 for a mutated S7 that encodes the amino acid substitutions responsible for converting VP7 to a soluble protein, i.e., P276H; R328A; V333N; A334P; P335M; V336P; and Q338P [42].

The AHSV4 LP strain is an attenuated virus; it is avirulent in horses and has reduced CPE in mammalian cells. Therefore, we included analysis of a highly virulent OIE reference strain of AHSV5 (HS 30/62), also known as “Fourie” or FR [73], to allow us to potentially gain more insight on the impact of VP7 solubility on AHSV virulence and/or cellular pathogenicity. We generated recombinant AHSV5 (rAHSV5) and a recombinant AHSV5 which contains a mutated S7 that expresses soluble VP7 (rAHSV5-sVP7) using reverse genetics technology as described above.

**Figure 1:**
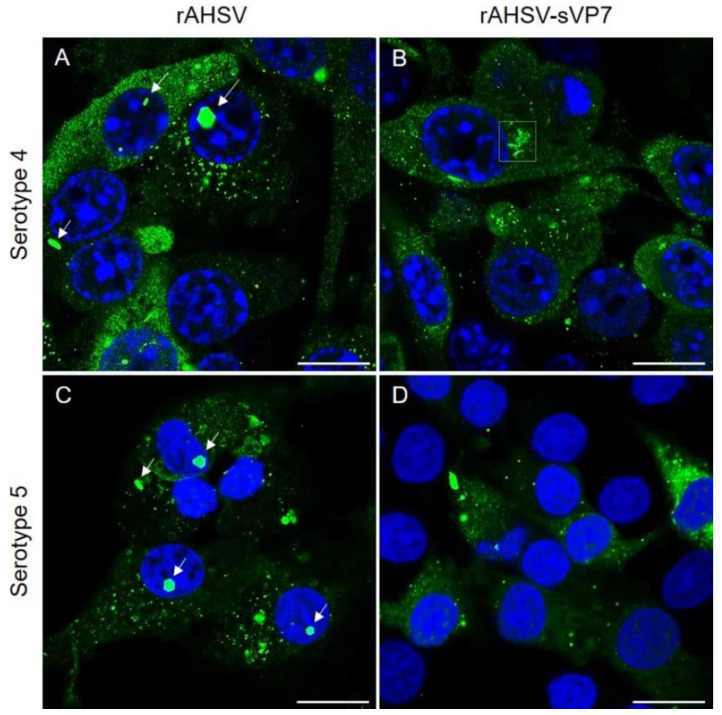
Intracellular VP7 distribution of recombinant reverse genetics-derived wild-type and mutant rAHSV of serotype 4 (**A**,**B**) and serotype 5 (**C**,**D**), respectively. Arrows indicate characteristic wild-type VP7 crystalline particles. Block indicates uncharacteristic VP7 tubules. BSR cell monolayers, seeded on coverslips, were infected at a MOI of 0.1 of each virus, incubated for 48 h, fixed and labelled as described in Section 2.5. VP7 was labelled with anti-VP7 guinea pig primary antibody and detected using Alexa Fluor 488 anti-guinea pig secondary antibody (green). Nuclei were stained with DAPI (blue). Scale bars represent 10 µm.

Successful virus rescue from transfection was confirmed by the presence of CPE, positive labelling of AHSV proteins and titration determination of passaged viruses. Furthermore, growth curves of rescued rAHSV and rAHSV5 and their respective wild-type reference strains AHSV4 (LP) [74] and AHSV5 (FR) [75] were revealed to be similar (Figure S1), confirming that these recombinant wild-type viruses fully represent their reference wild-type strains. To confirm the successful rescue of rAHSV that contains soluble VP7, the intracellular distribution of VP7 was compared between wild-type rAHSV and mutant rAHSV-sVP7 viruses. Infected BSR cells were fixed and labelled with anti-VP7 primary antibody and detected with secondary antibody by LSCM. As expected, characteristic VP7 crystals were observed for both rAHSV wild-type viruses (serotype 4 and 5) (Figure 1A,C, white arrows) while no such crystals were observed for rAHSV-sVP7 in either serotype 4 or 5 (Figure 1B,D). Small VP7 foci were present for wild-type and mutant rAHSV and rAHSV-sVP7 viruses. Interestingly, rAHSV4-sVP7-infected cells were observed to have numerous uncharacteristic tubule-like structures detected by anti-VP7 labelling within some cells (Figure 1B, white block). The distribution of soluble VP7 in rAHSV5-sVP7 appeared more homogenous, i.e., with less foci than rAHSV4, with some globular foci and no formation of tubule-like structures as seen for rAHSV4-sVP7 (Figure 1D).

The detection of virus proteins following rescue and after multiple passage events confirmed the ability of the mutant rAHSV4-sVP7 and rAHSV5-sVP7 viruses to replicate, indicating that VP7 crystalline particle formation is not vital for virus fitness.

### 3.2. Examining the Localisation of Soluble AHSV VP7 with Other Virus Proteins during Replication

As observed previously, when expressed alone, sVP7 has a homogenous intracellular distribution [42]. Therefore, any non-homogenous distribution of sVP7 as seen in Figure 1 can be assumed to be as a result of interaction with other virus proteins. Results thus far revealed that wild-type VP7 of both rAHSV4 and rAHSV5 had a similar distribution, i.e., hexagonal crystalline particle formation and small VP7 foci. Apart from the absence of VP7 crystalline particles, soluble VP7 of both mutant rAHSV4-sVP7 and rAHSV5-sVP7 also formed small VP7 foci, but rAHSV4-sVP7 additionally formed sVP7 tubule-like structures. To further explain these observations, the colocalisation of VP7 with other virus proteins was compared in rAHSV4 and rAHSV4-sVP7 infected cells. Slides were prepared as above and labelled with primary antibodies against different AHSV virus proteins and detected with the appropriate secondary antibodies.

**Figure 2:**
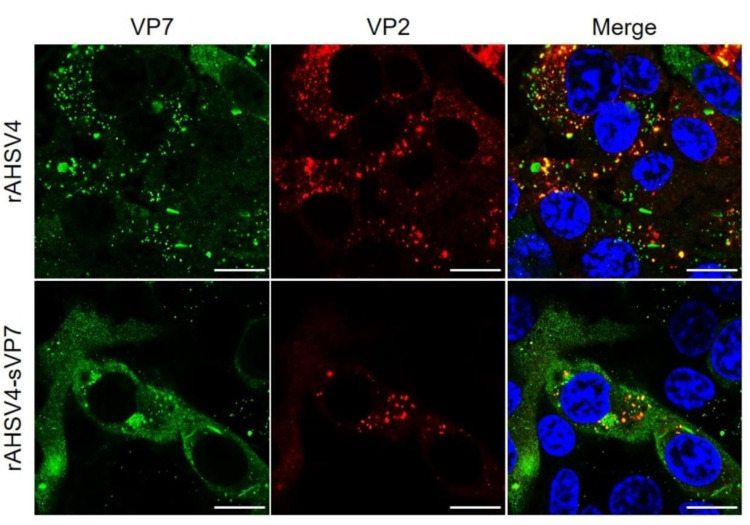
Intracellular distribution and colocalisation of AHSV VP7 and outer capsid protein VP2 in wild-type rAHSV4 (top panel) and mutant rAHSV4-sVP7 (bottom panel). BSR cell monolayers, seeded on coverslips, were infected at a MOI of 0.1 of each virus, incubated for 48 h, fixed and labelled as described in Section 2.5. VP7 was labelled with anti-VP7 guinea pig primary antibody and detected using Alexa Fluor 488 anti-guinea pig secondary antibody (green). VP2 was labelled with anti-VP2 mouse primary antibody and detected using Alexa Fluor 594 anti-mouse secondary antibody (red). Nuclei were stained with DAPI (blue). Images are split into separate channels (green and red) with channels merged on the right. Areas of colocalisation are indicated by yellow in merged images. Scale bars represent 10 µm.

The small VP7 foci observed to be present for wild-type and mutant viruses in Figure 1 were hypothesized to be VP7 localisation to the AHSV virion in the final stages of the AHSV replication cycle. To compare the association of the VP7 proteins with the AHSV virion, the colocalisation of VP7 with the outer capsid protein VP2 was analysed in rAHSV4 and rAHSV4-sVP7 infected cells. Results showed that the small VP7 foci observed in both mutant and wild-type viruses were colocalised with VP2 (Figure 2 and Figure S2). These results confirm that these foci are sites where VP2 likely assembles onto the core particle to form complete virions and/or are sites of cytoplasmic accumulation of complete virions prior to virus egress. Also noted was the absence of colocalisation of VP2 with wild-type VP7 crystalline particles or mutant sVP7 tubule-like structures. Interestingly, results indicated that less VP7-VP2 foci were present for mutant rAHSV4-sVP7 (Figure 2 and Figure S2).

**Figure 3:**
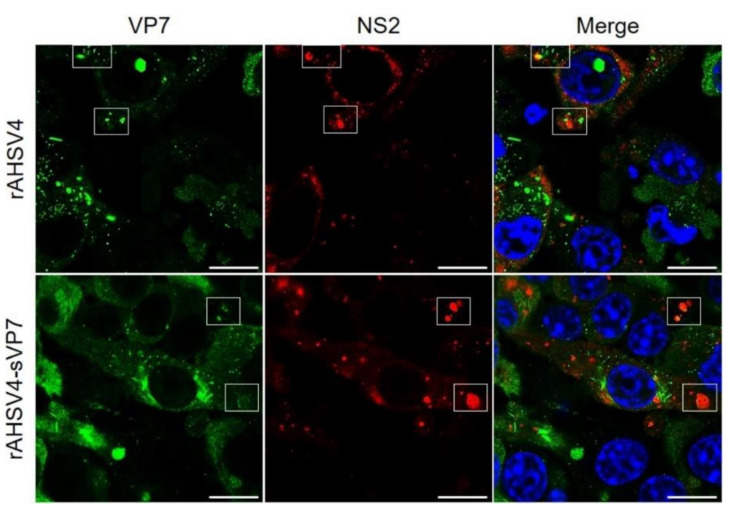
Intracellular distribution of AHSV VP7 with viral inclusion body mediator protein NS2 in wild-type rAHSV4 (top panel) and mutant rAHSV4-sVP7 (bottom panel). Boxes indicate VP7 foci association alongside VIBs. BSR cell monolayers, seeded on coverslips, were infected at a MOI of 0.1 of each virus, incubated for 48 h, fixed and labelled as described in Section 2.5. VP7 was labelled with anti-VP7 guinea pig primary antibody and detected using Alexa Fluor 488 anti-guinea pig secondary antibody (green). NS2 was labelled with anti-NS2 rabbit primary antibody and detected using Alexa Fluor 647 anti-rabbit secondary antibody (red). Nuclei were stained with DAPI (blue). Images are split into separate channels (green and red) with channels merged on the right. Areas of colocalisation are indicated by yellow in merged images. Scale bars represent 10 µm.

To compare the replication properties of wild-type and mutant rAHSV, next we examined the association of VP7 with VIBs. VIBs are virus factories which act as a scaffold for viral replication and assembly. VP7 foci have previously been shown to associate alongside VIBs [70]. To compare the localisation of VP7 with VIBs for rAHSV4 and rAHSV4-sVP7, the localisation of VP7 with the VIB mediator protein, NS2, was analysed (Figure 3). Results showed that both VP7 and sVP7 associate alongside VIBs in a similar manner (Figure 3 and Figure S3, white boxes) in rAHSV4 and rAHSV4-sVP7 infected cells, respectively.

**Figure 4:**
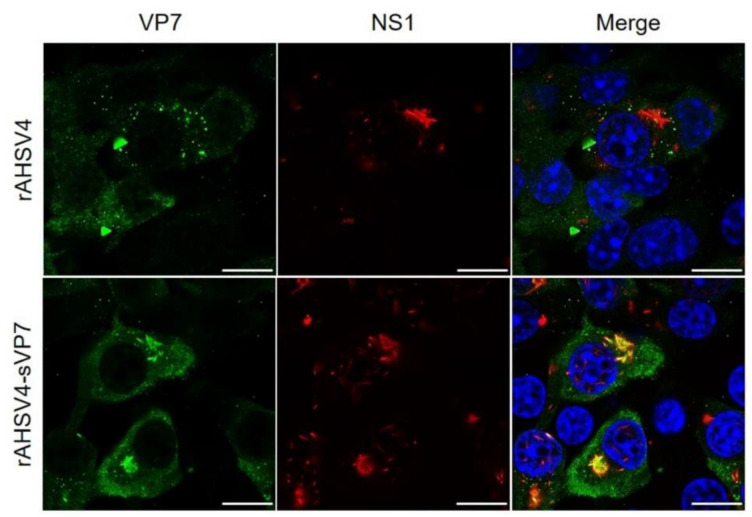
Intracellular distribution and colocalisation of AHSV VP7 with AHSV tubule-forming protein NS1 in wild-type rAHSV4 (top panel) and mutant rAHSV4-sVP7 (bottom panel). BSR cell monolayers, seeded on coverslips, were infected at a MOI of 0.1 of each virus, incubated for 48 h, fixed and labelled as described in Section 2.5. VP7 was labelled with anti-VP7 guinea pig primary antibody and detected using Alexa Fluor 488 anti-guinea pig secondary antibody (green). NS1 was labelled with anti-NS1 mouse primary antibody and detected using Alexa Fluor 594 anti-mouse secondary antibody (red). Nuclei were stained with DAPI (blue). Images are split into separate channels (green and red) with channels merged on the right. Areas of colocalisation are indicated by yellow in merged images. Scale bars represent 10 µm.

Results thus far indicated the presence of unique sVP7 tubule-like structures in rAHSV4-sVP7 LSCM data. To determine if the tubule-like distribution was due to association with characteristic AHSV NS1 tubules, dual labelling of VP7 and the tubule-forming NS1 protein was performed and analysed. Results showed that the tubule-like structures formed by sVP7 fully colocalised with the NS1 signal (Figure 4 and Figure S4), indicating a sVP7-NS1 association in rAHSV4-sVP7 infected cells. Interestingly, there was no wild-type VP7-NS1 colocalisation nor any redistribution of VP7 into tubule-like structures observed for wild-type rAHSV4.

Taken together, results thus far show that soluble VP7 associates with VP2 and NS2 proteins similarly to wild-type VP7. The notable difference observed can be seen for the unique colocalisation and potential interaction of soluble VP7 with NS1 tubules.

### 3.3. Examining the Role of AHSV VP7 Crystalline Particles in Virus Replication

To determine the impact of VP7 solubility on AHSV replication, in vitro time trials were performed in mammalian cells to determine the replication kinetics of each virus, i.e., attenuated (rAHSV4 versus rAHSV4-sVP7) and virulent (rAHSV5 versus rAHSV5-sVP7). To obtain a clear picture of the virus replication kinetics, released versus cell-associated virus was determined, in triplicate, at 12 hourly intervals over 3 days for each virus. Virus titre means were plotted and represented on a logarithmic scale to illustrate growth curves. Total virus was reported from the sum of titres from released and cell-associated fractions and illustrated to scale to show proportional differences between each virus at each time point.

**Figure 5:**
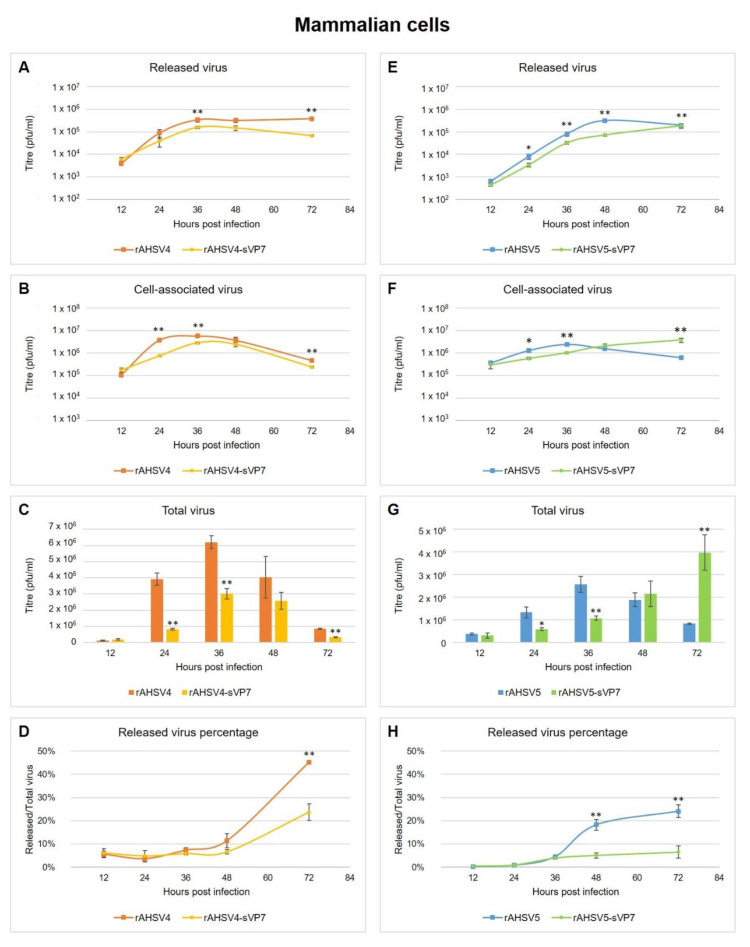
Replication kinetics of rAHSV and rAHSV-sVP7 recombinant viruses for attenuated serotype 4 (**left**) and virulent serotype 5 (**right**) in mammalian cells. (**A**,**E**) Released virus fraction from infected cells (mean titre of virus collected from media) represented on a logarithmic scale. (**B**,**F**) Cell-associated virus fraction from infected cells (mean titre of virus collected from lysed cell monolayers) represented on a logarithmic scale. (**C**,**G**) Total virus yield (sum of released and cell-associated virus fraction titres) represented on a linear scale to show proportional differences between each virus at each time point. (**D**,**H**) Percentage virus released (mean titre of released virus/mean titre of total virus*100). Equal amounts of BSR cells were infected at a MOI of 0.1 of each virus, in triplicate, and incubated for 12, 24, 36, 48 and 72 h. Media and cells were collected separately for titration as described in Section 2.7. Standard error of the mean is indicated and statistically significant differences to the wild-type virus is indicated as * (*p* ≤ 0.05) or ** (*p* ≤ 0.01).

Released virus titre was determined from collecting media separately from cell monolayers prior to cell lysis. Results showed that rAHSV4-sVP7 released significantly less virus throughout the 3-day period, culminating with a decreased yield of released progeny virus at 72 hpi (Figure 5A). Cell-associated virus titre was determined by collecting and lysing cell monolayers subsequent to media collection. Results indicated that virus yield was also significantly reduced in cell-associated fractions for rAHSV4-sVP7 throughout the 3-day period and especially at 24 and 36 hpi (Figure 5B). A decline in cell-associated virus yield is expected at later hours (48 and 72 hpi) for AHSV as overall cell death increases.

Looking at the overall wild-type rAHSV4 replication kinetics (Figure 5C), total virus yield amplified substantially from 12 hpi to 24 hpi and continued to peak at 36 hpi, after which virus yield begins to taper down steadily from 36 hpi to 72 hpi. This is consistent with experimental data where CPE is typically observed in AHSV-infected mammalian cells from 24 to 36 hpi. When comparing the resulting proportional differences in total virus yield for each serotype 4 virus (Figure 5C), it is observed that rAHSV4-sVP7 has an overall delayed replication rate compared to wild-type evidenced by significantly lowered overall yields throughout the 3-day interval (Figure 5C). Specifically, although both viruses start with similar initial infection rates at 12 hpi, rAHSV4-sVP7 replicates to five times lower titre at 24 hpi and peaks at half the total virus yield of rAHSV4 at 36 hpi (Figure 5C). The decrease in total virus yield at these times is due to reduced cell-associated virus production at 24 and 36 hpi (Figure 5B). Total virus yield culminates with three times lower yield than rAHSV4 at 72 hpi (Figure 5C). This decreased total virus yield at 72 hpi is due to the significant reduction in rAHSV4-sVP7 release at 72hpi (Figure 5A).

Percentage of released progeny virus, calculated as percentage of released fraction over total virus yield, indicated that rAHSV4 virus release increased rapidly from 36 to 72 hpi (Figure 5D), while rAHSV4-sVP7 has significantly less virus released from 36 hpi onward culminating in almost half released progeny virus overall (Figure 5D). Overall results for rAHSV4 and rAHSV4-sVP7 replication kinetics revealed that rAHSV4-sVP7 produced much less total virus than rAHSV4 with a significant reduction in intracellular virus production at peaks replication times and an overall reduction in released progeny virus. These findings coincide with the finding of reduced virions observed for rAHSV4-sVP7 previously (Figure 2).

Similarly to rAHSV4-sVP7, the released fraction of the virulent strain rAHSV5-sVP7 also had reduced virus titres over the 72 h period compared to rAHSV5 (Figure 5E). Interestingly, cell-associated virus titres of rAHSV5-sVP7 demonstrated a continued steady increase over the 72 h period ending with a significantly higher load of cell-associated virus than rAHSV5 by 72 hpi (Figure 5F). When comparing the total virus yield, it was found that rAHSV5 had similar growth kinetics to rAHSV4, in that virus production peaked at 36 hpi (Figure 5G) followed by a continuous decline in total virus yield. In contrast, it was found that rAHSV5-sVP7 titres increased exponentially from 12 to 72 hpi ending with a significantly increased total virus yield by 72 h compared to rAHSV5 (Figure 5G). No decline in rAHSV5-sVP7 virus production was observed, indicating that CPE does not occur with rAHSV5-sVP7 as it does with wild-type rAHSV5 or rAHSV4 and even rAHSV4-sVP7. Despite having similar initial replication rate than rAHSV5 at 12 hpi and a slower growth rate than rAHSV5 at 24 to 36 hpi, rAHSV5-sVP7 culminated in five times higher yield than rAHSV5 by 72 hpi (Figure 5G). Compared to rAHSV5, rAHSV5-sVP7 had a very low percentage of released progeny virus overall over the 72 h period and resulted in significantly less overall progeny virus released than rAHSV5 (Figure 5H). These results indicate that rAHSV5-sVP7 has increased intracellular progeny virus production over rAHSV5, yet less virus is released overall. The observed increase in cell-associated virus production after 48 hpi is likely due to lack of CPE in rAHSV5-sVP7-infected cells allowing for the continuous production of virus over this period.

Comparing attenuated versus virulent serotypes overall revealed that both rAHSV4-sVP7 and rAHSV5-sVP7 had a general reduction in virus release compared to their wild-type virus counterparts (Figure 5D,H). Interestingly, a major difference between the serotypes was in the production of progeny virus within the cell. Compared to wild-type, rAHSV5-sVP7 had a significant increase in cell-associated virus production after 48 hpi which attributed to a significant increase in total virus yield at 72 hpi (Figure 5F,G), versus rAHSV4-sVP7 which had a significant decrease in cell-associated virus production resulting in a significant decrease in total virus yield overall (Figure 5B,C). Therefore, these results suggest that VP7 crystalline particles play a role in virus release and, in the case of virulent serotype 5, AHSV-induced CPE and cell death.

Since AHSV is a vector-borne disease, it was decided to repeat this part of the study in the insect vector cell line (KC cells) to determine if VP7 solubility affects replication differently in these cells. Time trials were conducted as above, except the released and cell-associated virus fractions could not be collected separately as the cell integrity of the fragile KC cells could not be guaranteed at time of collection. Instead, at time of virus harvest, total virus was collected and titred as described in Section 2.7.

**Figure 6:**
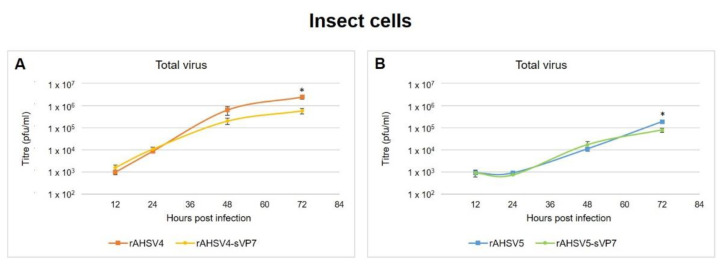
Replication kinetics of rAHSV and rAHSV-sVP7 viruses for attenuated serotype 4 (**A**) and virulent serotype 5 (**B**) in insect cells. Total virus yield from infected KC cells (mean titre of virus collected from media and cells) represented on a logarithmic scale. Equal amounts of KC cells were infected at a MOI of 0.1 of each virus, in triplicate, and incubated for 12, 24, 48 and 72 h. Media and cells were collected, lysed and titred as described in Section 2.7. Standard error of the mean is indicated and statistically significant differences to the wild-type virus is indicated as * (*p* ≤ 0.05).

In KC cells, both rAHSV4 and rAHSV5 wild-type virus titres increased continuously from 12 to 72 hpi (Figure 6) with no decrease in virus production as that observed in mammalian cells (Figure 5C,G). This is likely attributed to the lack of CPE observed for AHSV-infected insect cells [22,76]. It was found that both rAHSV4-sVP7 and rAHSV5-sVP7 replicated similarly to their wild-type counterparts from 12 to 48 hpi, but culminated in lower total virus yield by 72 hpi, i.e., four times lower yield for rAHSV4-sVP7 (Figure 6A) and two times lower yield for rAHSV5-sVP7 (Figure 6B). These results indicate that VP7 crystalline particles do not play a significant role in virus replication in insect cells but may be required for efficient virus production at later times post infection.

## 4. Discussion

Insoluble aggregates or inclusions that are formed during virus infection have been evidenced to have various functions within the cell [43]. For example, viruses can use aggregates to promote viral replication, gene expression and transport [43,77]. Protein aggregates can also cause host cell death by inducing ER stress and the unfolded protein response [54]. In addition, viruses can utilize these infection-associated protein aggregates to form protective structures that facilitate viral escape from cellular degradation machinery [43,77]. AHSV VP7 crystalline particles have previously been shown to form independently from host-virus interactions [70], and instead result from the intrinsic ability of the protein to self-assemble into these highly ordered crystalline lattices on its own [42].

This study aimed to understand the nature of the implied effects of crystalline particle formation on cellular biology and AHSV replication. A reverse genetics approach was used to generate a recombinant AHSV that contains a mutated segment 7 that encodes the soluble VP7 in its genome and expresses soluble VP7 during replication. Successful rescue of rAHSV-sVP7 for both attenuated and virulent strains confirmed that VP7 crystalline particle formation is not required for successful virus replication. The impact of this genetic knock-in of a soluble VP7 was analysed by observing virus protein-protein interactions and replication kinetics compared to wild-type rAHSV.

The soluble VP7 protein interacted similarly to wild-type VP7 with outer capsid protein VP2 and VIB mediator NS2 in infected cells, indicating that sVP7 forms cores as usual during replication. This served to confirm that wild-type VP7 is required in its soluble form to assemble into cores during replication as previously suggested [42,70]. Interestingly, sVP7 uncharacteristically colocalised with NS1 tubules in rAHSV4-sVP7 infected cells. No such sVP7 tubules were observed for rAHSV5-sVP7. While the function of AHSV NS1 tubules is unknown, NS1 could be interacting with excess sVP7 to sequester surplus virus protein during replication. Interestingly, a mutated form of NS3 has previously been shown to also uncharacteristically colocalise with NS1 tubules in the same manner observed here [31]. In addition, it was found that during infection with a NS4 knockout rAHSV5, NS1 colocalised more extensively with promyelocytic leukaemia nuclear bodies than NS1 in wild-type virus infected cells [66]. Therefore, alteration of other viral proteins tends to impact NS1 behaviour. While the role of NS1 tubules remains unknown, together these results further indicate the potential role of NS1 to possibly be a regulator of aberrant or misfolded viral proteins during replication. A previous study has reported that VP7 crystalline particles are occasionally associated with NS1 tubules in AHSV-infected KC cells [22]. NS1 of BTV has been shown to upregulate gene expression of virus proteins [18] which was later attributed to a non-tubule-forming version of NS1 [78]. Perhaps this typically rare VP7-NS1 association is enhanced in rAHSV4-sVP7, contributing to its characteristics observed here.

Previous studies have shown that increasing the solubility of AHSV VP7 increases the yield of CLPs [79]—likely due to the increased availability of VP7 that is no longer aggregated into crystalline particles [42]. It is therefore hypothesised that a recombinant AHSV that expresses a soluble VP7 could have increased virus production. With the availability of recombinant wild-type AHSV and AHSV-sVP7 viruses, we set out to test this hypothesis and to determine whether VP7 crystalline particles play a role in AHSV replication. Generally, the effect of a phenomenon on replication can be measured in terms of virus yield and virus release (replication kinetics) which, in turn, can contribute toward the outcome of cellular pathogenicity. Thus, the replication kinetics between rAHSV and rAHSV-sVP7 was compared for both attenuated and virulent strains.

Released and cell-associated virus fractions were collected to obtain a more detailed view of intracellular progeny virus production versus released virus. Initial infection rates were also compared by examining virus yield at 12 hpi. It was revealed that both wild-type and mutant viruses (serotypes 4 and 5) had similar initial replication rates and, following initial infection, differences in virus production and release were observed.

AHSV is typically released from the mammalian cell via disruption of the plasma membrane in a lytic manner mediated by NS3 [22,32]. When comparing the growth curves of wild-type rAHSV4 and rAHSV5 in BSR cells, both viruses display a rapid increase in virus production from 12 to 36 hpi followed by a gradual decline. This decline in cell-associated virus yield after 36 hpi is an indication of viral-associated cellular pathogenesis or CPE. Normally, AHSV infection of mammalian cells results in a severe cytopathic effect within 24 to 48 hpi manifested as cell rounding, apoptosis and lytic release of virions into the culture medium. Therefore, such a growth curve is expected in mammalian cells because as virus is produced, it is released over time in a lytic manner, resulting in cell death from extensive membrane damage. In contrast, no CPE is observed in insect cells due to non-lytic AHSV release mechanisms and absence of virus-induced apoptosis [22,76]. Therefore, AHSV growth in KC cells is expected to steadily increase over time without decline as virus is released in a non-lytic manner allowing continuous virus production as long as the environment accommodates cell growth. Here, we observed this expected steady increase with no decline for all recombinant viruses in KC cells.

When comparing rAHSV4 with rAHSV4-sVP7 replication kinetics in BSR cells, it was found that rAHSV4-sVP7 replicates slower than rAHSV4 with significantly reduced virus yield overall. The decreased total virus yield over 72 h was due to a decrease in intracellular virus production. Interestingly, there was also a significant reduction in rAHSV4-sVP7 release throughout the 72 h period. It is likely that the delay in virus replication observed for rAHSV4-sVP7 is due to reduced released progeny virus particles available for reinfection. VP7 crystalline particles therefore play a role in AHSV4 release and intracellular virus production in mammalian cells.

Replication kinetics of rAHSV5-sVP7 revealed that similarly to rAHSV4-sVP7, there was decreased virus release throughout the 72 h period. Therefore, VP7 crystalline particles also play a role in AHSV5 release in mammalian cells. Most noteworthy for serotype 5 was that although at early hours post-infection rAHSV5-sVP7 had lower yields than rAHSV5, instead of declining after 36 hpi the mutant virus continued to produce intracellular virus causing exponential growth from 48 hpi resulting in a significant increase in total virus yield at 72 hpi. The exponential growth of rAHSV5-sVP7 in BSR cells was similar to what is observed of AHSV growth in KC cells due to lack of cell death (CPE) as a result of non-lytic release mechanisms. It is therefore likely that, like KC cells, the infected BSR cells were not damaged by rAHSV5-sVP7 virus replication due to reduced CPE resulting in the increased total virus yield observed for rAHSV5-sVP7. For this virulent serotype, it is therefore possible that VP7 crystalline particles contribute toward cellular pathogenesis by disrupting the cellular membrane to aid virus release therefore causing CPE in mammalian cells. These results therefore implicate a potential role of AHSV VP7 crystalline particles in rAHSV5 cellular pathogenesis.

Replication kinetics of recombinant viruses in KC cells revealed similar results for both serotype 4 and 5. Since virulence factors would typically only play a role in host mammalian cells, it is expected that no notable differences be revealed in KC cells. The decreased virus yield of mutant viruses in KC cells at 72 hpi further indicates a potential role of VP7 crystalline particles in efficient intracellular virus production as observed for mutant viruses in mammalian cells. Since no CPE is observed for insect cells, VP7 crystalline particles are not required for non-lytic release therefore resulting in similar replication kinetics of wild-type and mutant AHSV4 and AHSV5 in KC cells observed here. Furthermore, virus replication kinetics in mammalian cells revealed that virus release was significantly reduced for both mutant viruses rAHSV4-sVP7 and rAHSV5-sVP7, further indicating that AHSV VP7 crystalline particles play a role in mammalian, i.e., lytic, virus release mechanisms for these strains.

Virus protein aggregates or structures that disrupt cellular membranes to promote pathogenesis and human disease have been previously reported. For instance, the accessory protein PB1-F2 of influenza A virus oligomerises into amyloid fibres, and has been shown to rupture cell membranes and cause cell death at late time points in human lung epithelial and monocyte cells [61,80]. As observed for rAHSV5-sVP7, a mutant ΔPB1-F2-virus showed no such cellular toxicity [60,80]. PB1-F2 amyloid supramolecular structures were concluded to likely be responsible for the lytic activity of PB1-F2 and the resulting membrane damage during influenza A infection [80].

Protein aggregates that mediate virus pathogenesis have also been shown for the nuclear export protein of a highly mutating H1N1 influenza A virus. This protein intrinsically forms aggregates which have been correlated with its role in virion budding [63,81]. Hepatis B virus (HBV) x protein (HBx) forms intracellular aggregates in the cytoplasm of HepG2 infected cells where it accumulates in large granules [82]. HBx protein aggregates have been shown to contribute to liver pathogenesis [83] and promote the progression of liver disease in HBV-infected patients [84].

Since no exponential growth of rAHSV4-sVP7 was observed, it would be suggested that VP7 crystalline particles do not contribute toward rAHSV4 cellular pathogenesis as they may for rAHSV5. The cellular pathogenicity mechanisms of these viruses differ considerably since rAHSV4 is an attenuated virus with lowered CPE in mammalian cells while rAHSV5 is a virulent virus and unattenuated in mammalian cells. Therefore the role of VP7 crystalline particles in each virus may differ. A possible explanation could arise from our LSCM data that revealed the presence of sVP7-NS1 tubules in rAHSV4-sVP7 infected cells. NS1 has previously been linked to virus morphogenesis and cellular pathogenesis [19,85]. Perhaps NS1 plays a role in the CPE observed during AHSV4 infection of mammalian cells and toward its in vivo attenuation.

## 5. Conclusions

This study has revealed that AHSV VP7 crystalline particles play a role in virus release during AHSV replication. Furthermore, AHSV VP7 crystalline particles are required for efficient intracellular virus production in mammalian and insect cells. It was further revealed that VP7 crystalline particles may play a role in the lytic release of AHSV5 which may contribute to cellular pathogenesis in mammalian cells. To confirm the role of AHSV VP7 crystalline particles in pathogenesis, further cell viability and in vivo studies should be performed. To further elucidate the role of VP7 crystalline particles in virus release, localisation analysis should be carried out with exit mediator protein NS3, and the association of NS1 with VP7 should be further explored in the context of virus production. This study advances the understanding of AHSV replication.

## Data Availability

The data generated and used in this study is available upon request from the corresponding author.

## Supplementary Materials

The following supporting information can be downloaded at: www.mdpi.com/article/10.3390/v14102193/s1, Figure S1: Virus growth comparison of wild-type (WT) AHSV and recombinant reverse genetics-derived AHSV for attenuated serotype 4 (left) and virulent serotype 5 (right) strains.; Figure S2: Further examples of the intracellular distribution and colocalisation of AHSV VP7 and outer capsid protein VP2 in wild-type rAHSV4 (left) and mutant rAHSV4-sVP7 (right).; Figure S3: Further examples of the intracellular distribution of AHSV VP7 with viral inclusion body mediator protein NS2 in wild-type rAHSV4 (left) and mutant rAHSV4-sVP7 (right).; Figure S4: Further examples of the intracellular distribution and colocalisation of AHSV VP7 with AHSV tubule-forming protein NS1 in wild-type rAHSV4 (left) and mutant rAHSV4-sVP7 (right).
