# Supplementary material for: Investigating the Role of African Horse Sickness Virus VP7 Protein Crystalline Particles on Virus Replication and Release
Source: Viruses. 2022 Oct 4;14(10):2193. doi: 10.3390/v14102193 (PMC9608501; doi:10.3390/v14102193)
Supplement: Supplementary file 1 [file viruses-14-02193-s001.zip › Figure S3.pdf]

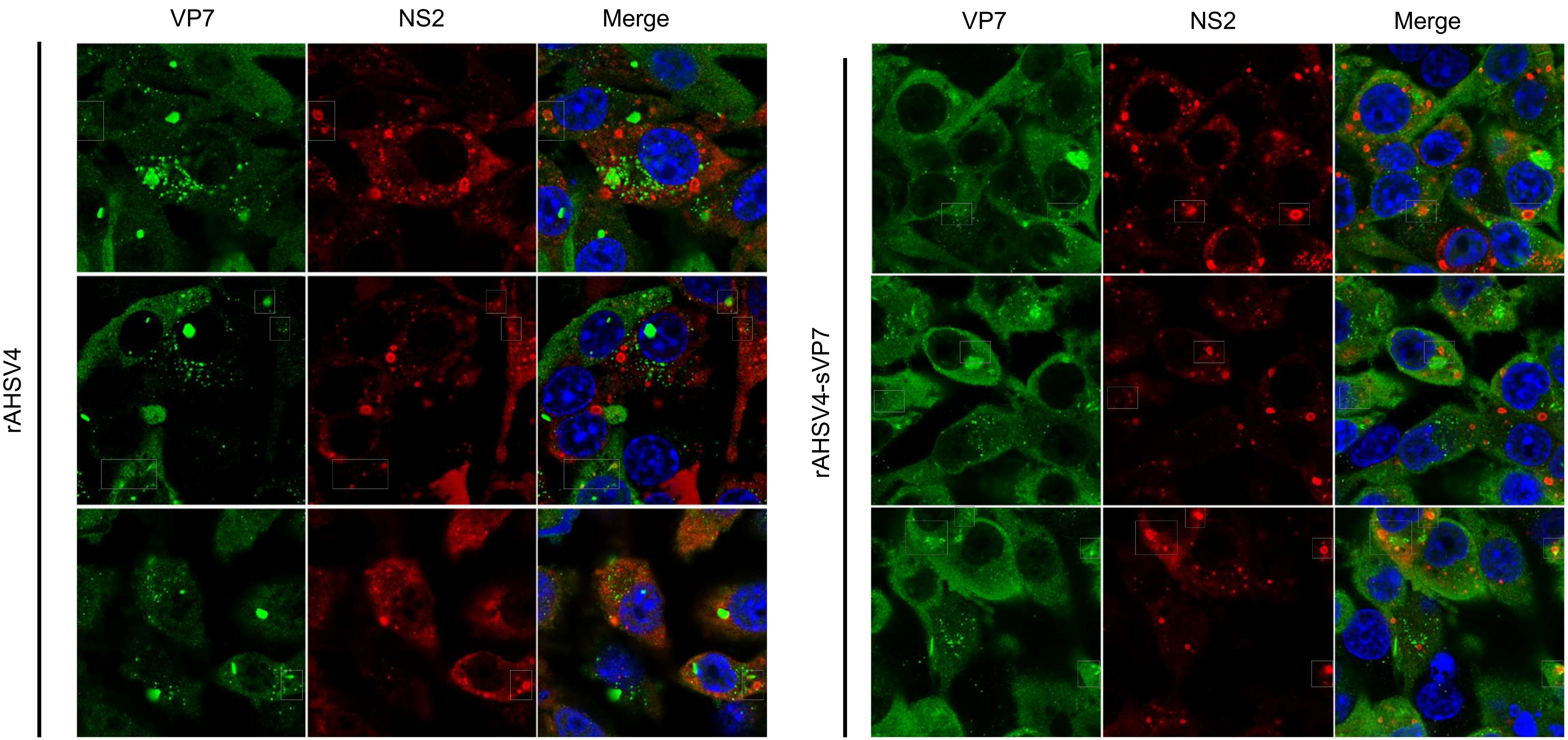

Figure S3. Further examples of the intracellular distribution of AHSV VP7 with viral inclusion body mediator protein NS2 in wild-type rAHSV4 (left) and mutant rAHSV4-sVP7 (right). Boxes indicate VP7 foci association alongside VIBs.
