# Supplementary material for: Investigating the Role of African Horse Sickness Virus VP7 Protein Crystalline Particles on Virus Replication and Release
Source: Viruses. 2022 Oct 4;14(10):2193. doi: 10.3390/v14102193 (PMC9608501; doi:10.3390/v14102193)
Supplement: Supplementary file 1 [file viruses-14-02193-s001.zip › Figure S1.pdf]

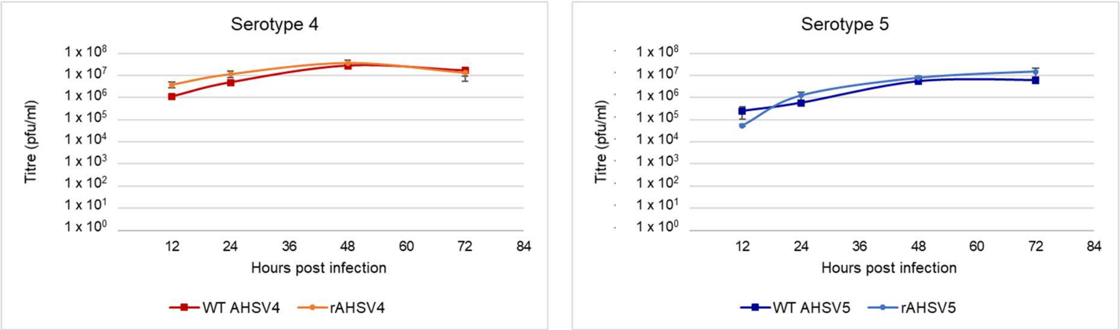

Figure S1. Virus growth comparison of wild-type (WT) AHSV and recombinant reverse genetics-derived AHSV for attenuated serotype 4 (left) and virulent serotype 5 (right) strains. The WT AHSV4 (“LP” isolate HS 32/62) and AHSV5 (“FR” isolate HS 30/62) are OIE reference strains that were available at the start of the study and previously described [73]. Total virus yield from infected BSR cells (mean titre of total virus) represented on a logarithmic scale. Standard error of the mean is indicated. Equal amounts of BSR cells were infected at a MOI of 0.1 for each virus, in triplicate, and incubated for 12, 24, 48, and 72 h. Media and cells were collected, lysed and titres were determined as described previously for AHSV4 [74] and AHSV5 [75]. No significant statistical difference between each virus was found.
